# Evaluation of the effectiveness of infrared light-emitting diode photobiomodulation in children with sleep bruxism: Study protocol for randomized clinical trial

**DOI:** 10.1097/MD.0000000000017193

**Published:** 2019-09-20

**Authors:** Fernanda Yukie Kobayashi, Paula Midori Castelo, Marcela Leticia Leal Gonçalves, Lara Janisky Motta, Ana Carolina da Costa Mota, Olga Maria Altavista, Marcelo Mendes Pinto, Monica Canuto Salgueiro, Kristianne Porta Santos Ferreira, Sandra Kalil Bussadori

**Affiliations:** a Departament of Biophotonics Applied in Health Science—UNINOVE, São Paulo; b Department of Pharmaceutical Sciences—Universidade Federal de São Paulo, (UNIFESP), Diadema; c Management in Health Systems, Nove de Julho University, São Paulo, Brazil.

**Keywords:** children, electromyography, LED, salivary biomarkers, sleep bruxism

## Abstract

**Background:**

Sleep bruxism is a masticatory muscle activity characterized as rhythmic (phasic) or nonrhythmic (tonic). In children and adolescents, etiological factors, such as breathing pattern and sleep quality, have recently been addressed in studies investigating sleep bruxism. New therapies for adults, such as botulinum toxin, have been investigated, but such techniques are not applicable for individuals in the growth and development phase.

**Methods:**

The participants will be 76 children, which will be randomly allocated to a control group, that is group 1, absence of bruxism; group 2, children with bruxism treated with infrared light-emitting diode (LED); and group 3, bruxism treated with occlusal splint. All participants will be submitted to a clinical evaluation to evaluate muscle activity and salivary biomarkers, before and after treatments. Muscle activity will be verified by electromyography of muscles mastication, masseter and temporal, and salivary biomarkers observed will be cortisol and dopamine levels.

**Discussion:**

Photobiomodulation therapy has piqued the interest of researchers, as this noninvasive method has demonstrated positive results in problems related to muscle tissues. This document describes the protocol for a proposed study to evaluate morphological and psychosocial aspects in children and adolescents with awake bruxism and their responses to photobiomodulation therapy with infrared LED.

**Clinical trials:**

https://clinicaltrials.gov/ct2/show/NCT03710174

## 1 Introduction

Sleep bruxism is a masticatory muscle activity during sleep that is characterized as rhythmic (phasic) or nonrhythmic (tonic) and is not a movement disorder or a sleep disorder in otherwise healthy individuals1. Clenching and/or grinding the teeth while sleeping is denominated primary or sleep bruxism, whereas clenching/grinding the teeth during the day is denominated secondary or awake bruxism.^[1]^ The etiology of bruxism is widely discussed in the literature. Studies investigating emotional disorders have increasingly found an association with parafunctional muscle activity, such as bruxism. According to Wieckiewicz et al (2014),^[2]^ bruxism may be related to the occlusion or may be caused entirely by psychological stimuli. Indeed, individuals with a more aggressive, compulsive, or controlling profile are reported to be more susceptible to the development of bruxism.^[3]^

Stress can be measured using salivary biomarkers, such as cortisol, which is a hormone activated by the hypothalamus-pituitary-adrenal axis in response to a physical or psychological stressor. Repeated exposure to stress can trigger excessive cortisol secretion, which can have harmful effects on one's health.^[4]^ Dopamine is a neurotransmitter from the family of catecholamines and phenylethylamines that, among other functions, contributes to the control of movements, learning, mood, emotions, cognition, and memory.^[5]^ Like cortisol, dopamine can also be quantified based on salivary levels.^[6]^ Changes in dopamine levels have been related to neuropsychiatric disorders, such as Parkinson disease, in which diminished concentrations of dopamine are found in the nigrastriatal pathway, or schizophrenia, both of which are diseases that affect the motricity of muscles.^[6]^ Although some studies have evaluated the dopamine concentration in salivary fluid,^[5,6]^ no studies have investigated dopamine concentrations in patients with bruxism, despite the fact that this problem affects the motricity of oral muscles.

The surrounding environment exerts a direct influence on the reflexes of the body in response to a stressor. All impressions related to the external environment are processed and evaluated by the central nervous system, more precisely the limbic system and hypothalamus, which provoke adequate emotions and stimulate the sympathetic nervous system, releasing adrenaline, which triggers changes, such as increases in the respiratory rate, heart rate, muscle tension, glucose level, and blood pressure. Any information that provokes such a response is recognized as a stressor.^[7,8]^ The effects of the suppression of emotions and motor activities overload the function of the organism, resulting in various neuromuscular disorders.^[9]^

Clenching the teeth has been associated with anxious tendencies.^[10]^ Anxiety in children is a common occurrence in pediatric clinical practice. The prevalence of anxiety ranges from 2.5% to 5% in the general population and 10.6% to 24% in children with bruxism.^[11]^ Differently from adults, symptoms related to anxiety change with the different phases of development in children, which often makes identification difficult. Therefore, if not detected at an early age, bruxism could lead to consequences that compromise the function of the stomatognathic system, exerting a negative effect on quality of life.

The most indicated treatment for bruxism is an occlusal adjustment with the use of a bite plate (Michigan splint).^[12]^ Alternative treatments for muscle disorders have been developed and have demonstrated good results, such as low-level laser therapy (LLLT). LLLT has been administered to acupuncture points in adults with temporomandibular disorder,^[13]^ with a significant difference found in signs and symptoms of the disorder following application of the technique. The authors attribute the success of therapy to the reduction in pain and muscle relaxation promoted by LLLT. Physiologically, LLLT is a form of biostimulation involving the occurrence of increased blood circulation, vasodilatation, analgesia, an anti-inflammatory effect, a reduction in edema, and the acceleration of the healing process of injured tissues. Photobiomodulation therapy with a light-emitting diode (LED) has also been used for the treatment of muscle disorders. Silva et al. (2015)^[14]^ established a treatment protocol for fibromyalgia including the use of LED. Besides the physiological gains that occur with LLLT, the authors consider LED treatment accessible because of its low cost and more durable equipment, making this a viable treatment option.

## 2 Aim

The aim of this project is to establish a protocol for evaluation of effectiveness of photobiomodulation therapy in children with sleep bruxism.

## 3 Material and methods

This project was evaluated by the human research ethics committee of University Nove de Julho (certificate of approval: 1.333.636) and clinical trials ID NCT03710174. The sample size was calculated considering α = 0.05 and an 80% power (n = 16 per group), to which 20% will be added to compensate for possible dropouts (n = 19 per group), totaling 76 children and adolescents. Additionally, sample size was calculated for internal validity by using the results of a previous study that evaluated the decrease in signs and symptoms of TMD after occlusal splint therapy (Restrepo et al^[15]^). Considering a mean difference between treatments equal to 2 (SD = 2), 3 groups, power of test of 80%, and an alpha level of 0.05, a minimum of 30 subjects should be included in each group.

**Figure 1:**
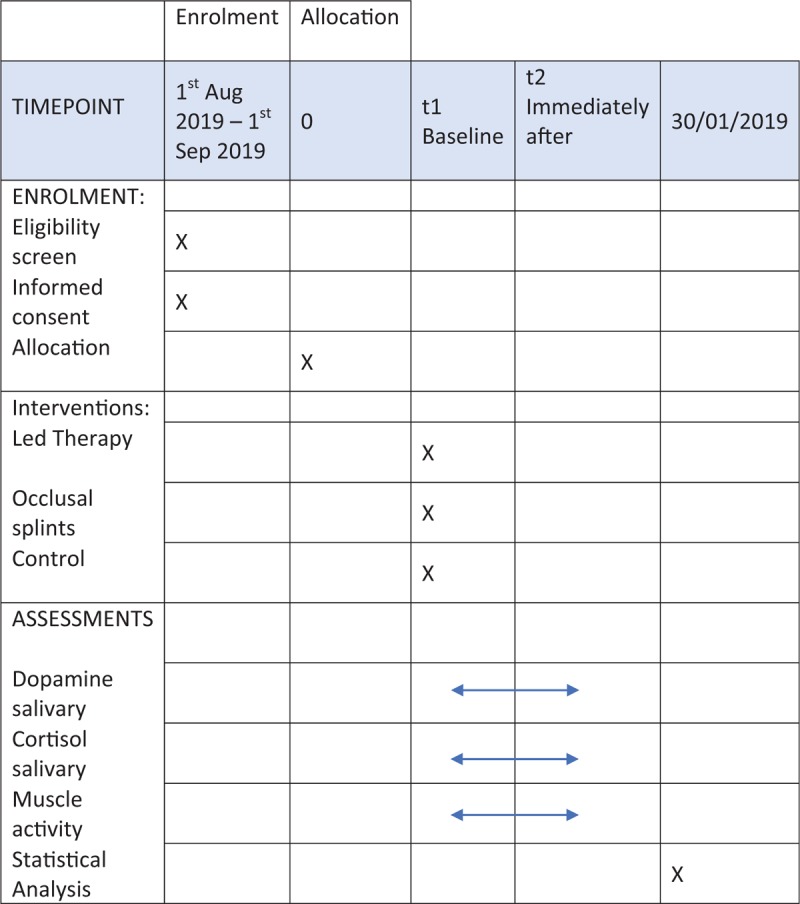
Standard protocol items: recommendations for interventional trials (SPIRIT) figure as recommended by 2013 SPIRIT statement.

**Figure 2:**
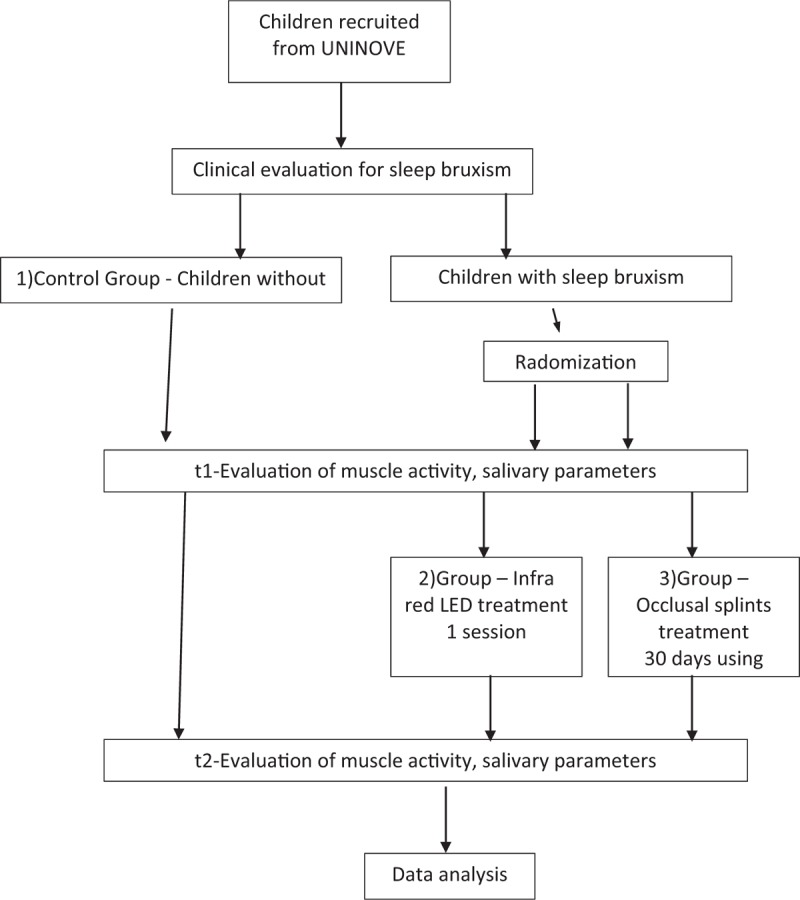
Flowchart activity.

The protocol is in accordance with the 2013 SPIRIT (Standard Protocol Items: Recommendations for Interventional Trials) Statement. The SPIRIT checklist can be found as an additional file and Figure 1 is the SPIRIT figure. SPIRIT was developed to provide guidance in the form of a checklist of recommended items to include in a clinical trial protocol, to help improve its content and quality (Fig. 2).

### 1.1 Inclusion criteria

The sample will be composed of children in the mixed dentition phase (permanent incisors and molars erupted) and adolescents with an established permanent dentition.

### 1.2 Exclusion criteria

Individuals with dental caries, those taking medications, such as anti-inflammatory agents, muscle relaxants, corticoids, anticonvulsants, and antidepressants, those with chronic diseases that affect muscles or motor coordination, and those who do not cooperate during the evaluation will be excluded from the study.

### 1.3 Allocation procedure

Block randomization (groups of 4 participants) will be performed using sequentially numbered (1–76) opaque envelopes. The contents of the envelopes will be determined randomly with the aid of a computer program. Each envelope will contain a piece of paper stipulating the group to which the child/adolescent will be allocated. The envelopes will be sealed until the time of treatment.

#### 1.3.1 Groups:

The participants will be randomly allocated to the following groups:

1. Group 1: control—absence of bruxism.
2. Group 2: bruxism treated with infrared LED.
3. Group 3: bruxism treated with occlusal splint.

### 1.4 Evaluation of bruxism

The diagnosis will be based on the reports of parents/caregivers regarding the occurrence of grinding the teeth. For such, parents/caregivers will receive a patient history questionnaire to fill out and deliver to the school. The following clinical signs will also be investigated for the diagnosis: abnormal tooth wear on functional cusps of the teeth, tooth marks on tongue, linea alba on the buccal mucosa along the occlusal plane, gingival recession, mandibular and/or maxillary torus, fractures, and/or cracks on the teeth.^[12]^

### 1.5 Photobiomodulation treatment protocol

The volunteers in Group 2 will be submitted to the initial evaluation of the morphological and psychosocial variables. During the same appointment, red LED (3 × 6 cm) will be administered using a board with 6 LEDs with a wavelength of 650 ± 20 nm, 7-minute operation time, optical spot of 5 ± 2 mm, and optical output of 2∼5 mW, with a dose of 2.675 J/cm^2^. Further analyses will be performed immediately after the photobiomodulation session and 1 week later. Group 3 will be submitted to the same analyses using an infrared LED protocol (wavelength: 850 ± 20 nm) with the same dose of 2.675 J/cm^2^.

The volunteers in Group 4 will be treated using the standard protocol of a rigid occlusal splint. After the initial evaluation, molds will be made for the fabrication of the splints, which will be delivered 1 week later. Written and verbal instructions for use will be given. After 1 month of daily use, the volunteers will return for the final morphological and psychosocial evaluations.

#### 3.5.1 Assessment aspects

The effectiveness of the photobiomodulation therapy will be assessed by the following protocols:

### 1.6 Protocol for electromyographic evaluation

Electromyography will be performed to complement the evaluation of the morphological aspects of the groups. The masseter and temporal muscles will be evaluated using a portable electromyograph (BTS TMJOINT) with wireless electrodes. The participant will be seated with Camper plane parallel to the floor. Three readings will be made on both sides with the muscles at rest, during habitual maximum intercuspation (isometric contraction), and during simulated chewing with Parafilm (isotonic contraction). The signal will be captured for 10 seconds under each condition. The first chewing cycle will be discarded and the subsequent 5 cycles will be collected.

### 1.7 Protocol for evaluation of salivary cortisol and dopamine levels

The participants and caregivers will receive verbal and written instructions to avoid any physical activity, the ingestion of substances with alcohol or caffeine, soft drinks, tea, corticoids, and chewing gum in the 24 hours before the collection of the saliva. Saliva samples will be collected using polyester swabs (Salivette, Sarstedt, Germany), which will be refrigerated immediately after collection. The swab will be placed under the tongue and the participant will be instructed to move it around the oral cavity until it becomes soaked with saliva (stimulated saliva). Samples with visible signs of blood will be discarded because of possible contamination by plasma cortisol (Miller et al, 1995). The swabs will be centrifuged at 3500 rpm for 5 minutes. The supernatant will be collected and stored at −40°C. Cortisol will be determined using an enzyme-linked immunosorbent assay (ELISA) (Salimetrics, State College, PA) at room temperature (25°C).

Dopamine will also be determined using an ELIZA kit (Dopamine Research ELISA BA E-5300). The samples will be thawed and centrifuged again. The sample volume will be 25 μL and incubation time will be 60 minutes. The plates will also have controls and standards. The procedure will follow the basic ELISA principle of competition between an untagged antigen and an enzyme-tagged antigen for a particular number of binding sites on the antibody. The analysis will involve reading the absorbance of the solution using a microplate reader set at 450 nm and 630-nm correction filter.

### 1.8 Analysis of results

Data will be statistically analyzed using SPSS 24.0 software (IBM Corp., NY), considering an alpha level of 5%, by one of the authors (PMC, Applied Statistics Specialist). The exploratory statistics will consist of means, standard deviation, medians, and quartiles. Normality will be tested by Shapiro–Wilk test and Quantilequantile-plot (QQ-plot) analysis. Those variables that do not show normal distribution will be transformed by the natural logarithm (ln).

A general linear model—2-way mixed model analysis of variance (ANOVA)—will be used to test the treatment effect in the observed variance of muscle activity and levels of salivary markers (considered as dependent variables). The effect size (partial Eta squared) and the power of the test for each model will also be obtained. The results of the Mauchley sphericity test and Levene equality of variances will be evaluated as ANOVA premises; when necessary, the Huynh-Feldt correction will be applied.

## 4 Discussion

There are some studies related to photobiomodulation in bruxism patients in the literature,^[13]^ but there are none with the use of the infrared LED as the light source, especially in bruxism.^[14]^ Clinically, the relevance of the study is given by the fact that LED therapy is much cheaper than laser, providing greater accessibility to the population.

## Author contributions

**Conceptualization:** Kristianne Porta Santos Ferreira, Sandra Kalil Bussadori.

**Formal analysis:** Paula Midori Castelo, Olga Maria Altavista.

**Funding acquisition:** Kristianne Porta Santos Ferreira.

**Investigation:** Marcela Leticia Leal Gonçalves, Leticia Leal Gonçalves, Ana Carolina da Costa Mota, Olga Maria Altavista.

**Methodology:** Fernanda Yukie Kobayashi, Ana Carolina da Costa Mota, Marcelo Mendes Pinto.

**Project administration:** Monica Canuto Salgueiro.

**Resources:** Monica Canuto Salgueiro.

**Software:** Marcela Leticia Leal Gonçalves

**Supervision:** Monica Canuto Salgueiro.

**Validation:** Marcela Leticia Leal Gonçalves, Monica Canuto Salgueiro, Sandra Kalil Bussadori.

**Visualization:** Ana Carolina da Costa Mota.

**Writing – original draft:** Fernanda Yukie Kobayashi, Marcela

**Writing – review & editing:** Paula Midori Castelo, Sandra Kalil Bussadori.
